# The tight-binding formulation of the Kronig-Penney model

**DOI:** 10.1038/s41598-017-17223-2

**Published:** 2017-12-06

**Authors:** F. Marsiglio, R. L. Pavelich

**Affiliations:** Department of Physics, University of Alberta, Edmonton, AB, T6G 2E1 Canada

## Abstract

Electronic band structure calculations are frequently parametrized in tight-binding form; the latter representation is then often used to study electron correlations. In this paper we provide a derivation of the tight-binding model that emerges from the exact solution of a particle bound in a periodic one-dimensional array of square well potentials. We derive the dispersion for such a model, and show that an effective next-nearest-neighbour hopping parameter is required for an accurate description. An electron-hole asymmetry is prevalent except in the extreme tight-binding limit, and emerges through a “next-nearest-neighbour” hopping term in the dispersion. We argue that this does not necessarily imply next-nearest-neighbour tunneling; this assertion is demonstrated by deriving the transition amplitudes for a two-state effective model that describes a double-well potential, which is a simplified precursor to the problem of a periodic array of potential wells. A next-nearest-neighbour tunneling parameter is required for an accurate description even though there are no such neighbours.

## Introduction

The notion of an “effective model” or “effective potential” pervades essentially all of physics. Even at the undergraduate level, for example, it is worth emphasizing that the lowly harmonic oscillator potential is really merely an “effective potential”. In reality all potentials are generally more complicated—the spring will eventually stretch inelastically—and any potential has a useful domain of applicability in every problem.

One can go further with “effective models”, with perhaps the best-known example being Feynman’s description of the ammonia molecule as a two-state system^1^. This sort of description is worthwhile for certain aspects of the problem, such as the time dependence of the wave function, but it often remains unclear how parameters required in the effective model are related to underlying “microscopic” characteristics of the same problem. A more concrete realization of the Feynman two-state system is the double-well potential. In ref.^2^ (see also ref.^3^) the states describing a particle in such a system were determined from the basic parameters, namely the barrier height and width. At the same time, a “toy model” describing a two-state system with a single parameter, a transition amplitude *t*, to describe tunneling through the barrier (analogous to the amplitude for the nitrogen atom to tunnel from below to above the plane of hydrogen atoms in the ammonia example), was shown to very accurately describe the ground state splitting calculated by solving the complete Schrödinger equation. This was an example of a case where the original model, with a Hilbert space consisting of an infinite number of states was approximately mapped onto a “toy” or “effective” model consisting of just two states.

In this paper we want to focus on a natural extension of this model, which occurs often in condensed matter, and leads to a “starting” model for studying strongly correlated electron systems. In this case we start with a periodic array of some potential that gives rise to energy bands, whose characteristics require a complete solution to the Schrödinger equation. This calculation in principle involves an infinite Hilbert space, in two senses. First, even a single potential well, representing a single atom with which an electron interacts, requires an infinite Hilbert space. However, for a solid there are a large number of these wells—an infinite number if we allow the solid to go on forever. This latter infinity is handled analytically through Bloch’s theorem^4–7^, which allows solution of the electron wave function in the infinite periodic array in terms of the solution within a single unit cell (see also ref.^8^ where some context for this theorem is provided). Even with Bloch’s theorem, however, an infinite Hilbert space is required to describe the (infinite) set of energy bands that emerges from the periodicity. An effective model, known as a tight-binding model, reduces the infinite Hilbert space down to an *N*-dimensional Hilbert space, where *N* is the number of atoms. This description is entirely analogous to the reduction of the double-well potential to a 2-dimensional Hilbert space, involving a single tunneling parameter *t*, and in fact, even as *N* → ∞, only a single parameter *t*, called the “tunneling amplitude”, remains.

This “down-folding” process is often carried out for complete descriptions of rather complicated systems, often with approximations or parameterizations along the way^9^. The purpose of this paper is to implement this procedure using the simplest model possible, i.e. the one-dimensional Kronig-Penney model^10^. The result is a derivation of the one or two parameters in the “effective” tight-binding model, in terms of the microscopic parameters that describe the original Kronig-Penney model. This calculation is possible because some aspects of the one-dimensional Kronig-Penney model are known analytically.

For reasons that will become clear as we proceed, comparisons are required with the double-well case (i.e. a Kronig-Penney model with only 2 cells). This solution, performed for a configuration pertinent to the infinite-cell Kronig-Penney model, is described in Appendix A. Our numerical solutions confirm that this description is exact in the limit of tightly-bound wells, and we explore further to what degree this description remains accurate as the coupling between wells increases. Remarkably, we find that the need for so-called next-nearest-neighbour (nnn) hopping does not necessarily imply next-nearest-neighbour hopping. This exercise, carried out here only for this specific model^10^, gives us a deeper understanding of the connection between effective models and their more microscopic counterparts.

These results complement the phenomenological fits realized in refs^2,7^. The first reference describes a double-well potential, and can be thought of as a special preliminary case of the fully periodic solid. This form of the double-well potential treated in this reference is not directly applicable to the present problem but, for completeness, we include its solution separately in Appendix B.

## Kronig-Penney Model

**Figure 1:**
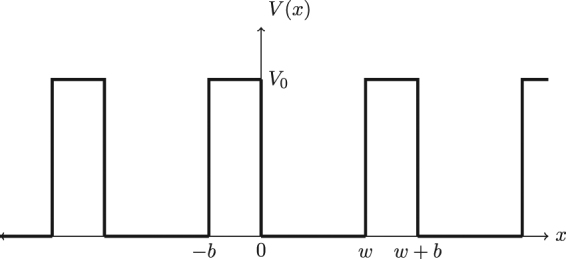
A pictorial representation of the periodic potential in the Kronig-Penney model, illustrating wells of depth *V*_0_ and width *w* separated from one another by barriers of width *b*.

The one-dimensional Kronig-Penney model^10^ consists of an electron moving in a periodic potential as depicted in Fig. 1, with alternating wells of width *w* and barriers of width *b* and height *V*_0_. The basic building block of this model is the unit cell, here consisting of one well surrounded by two (half) barriers; in the limit that the barrier heights and/or widths become large, a good starting point for this problem is the solution to the problem of a single well, and we will exploit this in what follows. However, the physics that emerges from the Kronig-Penney model is already contained in the double-well potential, whose solution is given in Appendix A (see also Appendix B for the solution to the double-well considered in ref.^2^),

Proceeding with the Kronig-Penney model, the analytical solution for the energy levels (*E* < *V*_0_) is well known; the implicit equation for the energy is

1 $$\cos (k\ell )=\,\cos (qw)\,\cosh \,({\kappa }_{2}b)+\frac{{\kappa }_{2}^{2}-{q}^{2}}{2q{\kappa }_{2}}\,\sin \,(qw)\,\sinh \,({\kappa }_{2}b),$$

where $\ell =w+b$ is the unit cell length, and $q=\sqrt{2mE/{\hslash }^{2}}$ and ${\kappa }_{2}=\sqrt{2m({V}_{0}-E)/{\hslash }^{2}}$. For each wave vector *k*, with values −$\pi  < k\ell \le \pi$, one needs to solve this equation for *E*(*k*). A similar equation holds for *E* > *V*_0_ (replace *κ*_2_ → *ik*_2_, where ${k}_{2}\equiv \sqrt{2m(E-{V}_{0})/{\hslash }^{2}}$). As is well known, the periodicity in the problem gives rise to a series of energy bands as a function of wave vector *k*, with each band separated by an energy gap. In the case where the wells illustrated in Fig. 1 are deep, any single well, taken in isolation, would consist of a number of different energy levels corresponding to states that are bound within each well. As already stated, when *N* of these wells are coupled through barriers, each of these energy levels broadens into *N* states, forming bands. Numerical solutions to this and other periodic models with different potential shapes are given in refs^6^ and^7^.

## Tight-Binding

The tight-binding limit tends to focus on one of these bands, and is used to describe the dispersion, *E*(*k*) for this band. General considerations^5^ in the tight-binding limit in one dimension lead to a dispersion of the form

2 $$E(k)={E}_{b}-2{t}_{1}\,\cos (k\ell )-2{t}_{2}\,\cos \,\mathrm{(2}k\ell )-2{t}_{3}\,\cos \,\mathrm{(3}k\ell )-\ldots $$

The usual interpretation of such a dispersion is that each additional term corresponds to tunneling of an electron from a well to a further neighbouring well. In other words, while *t*_1_ represents a tunneling amplitude for an electron to tunnel through one of the barriers in Fig. 1, *t*_2_ represents a tunneling amplitude for the electron to tunnel through two of the barriers, and end up (directly) two unit cells away from its initial location. Given that the electron wavefunctions are exponentially decaying in the barrier regions, it should be clear that $|{t}_{1}|\gg |{t}_{2}|\gg |{t}_{3}|\gg \ldots$ in this limit. In what follows we will first focus on nearest-neighbour tunneling amplitudes only, i.e. we will obtain from Eq. (1), an explicit expression for *t*_1_.

Motivated by the case when *V*_0_ or *b* is suitably large, one can rewrite Eq. (1) to obtain, without approximation,

3 $$(\cos \,\frac{qw}{2}-\frac{q}{{\kappa }_{2}}\,\sin \,\frac{qw}{2})\,(\cos \,\frac{qw}{2}+\frac{{\kappa }_{2}}{q}\,\sin \,\frac{qw}{2})={\eta }_{1}(k)+{\eta }_{2}$$

where

4 $$\begin{array}{rcl}{\eta }_{1}(k) & = & 2\,{e}^{-{\kappa }_{2}b}\,\cos \,(k\ell )\\ {\eta }_{2} & = & -{e}^{-2{\kappa }_{2}b}\,(\cos \,\frac{qw}{2}-\frac{{\kappa }_{2}}{q}\,\sin \,\frac{qw}{2})\,(\cos \,\frac{qw}{2}+\frac{q}{{\kappa }_{2}}\,\sin \,\frac{qw}{2}).\end{array}$$

Written in this way, it is easy to see that when there is no coupling between the wells (e.g. put *b* → ∞) and therefore both *η*_1_(*k*) → 0 and *η*_2_ → 0, then the vanishing of the first (second) factor on the LHS of Eq. (3) corresponds to determining the energy for the even (odd) bound states in the single well. It is convenient to define dimensionless variables as before, specifically *z* ≡ *qw*/2 and *z*_0_ ≡ *k*_0_ *w*/2, where ${k}_{0}\equiv \sqrt{2m{V}_{0}/{\hslash }^{2}}$. Then Eq. (3) reads

5 $$(\cos \,z-\frac{z}{\sqrt{{z}_{0}^{2}-{z}^{2}}}\,\sin \,z)\,(\cos \,z+\frac{\sqrt{{z}_{0}^{2}-{z}^{2}}}{z}\,\sin \,z)={\eta }_{1}(k)+{\eta }_{2},$$

where we have used ${\kappa }_{2}w/2=\sqrt{{z}_{0}^{2}-{z}^{2}}$ and now

6 $${\eta }_{1}(k)=2{e}^{-2\tfrac{b}{w}\sqrt{{z}_{0}^{2}-{\tilde{z}}_{1}^{2}}}\,\cos \,k\ell $$

and

7 $${\eta }_{2}={e}^{-4\tfrac{b}{w}\sqrt{{z}_{0}^{2}-{\tilde{z}}_{1}^{2}}}\,(\cos \,z-\frac{\sqrt{{z}_{0}^{2}-{z}^{2}}}{z}\,\sin \,z)\,(\cos \,z+\frac{z}{\sqrt{{z}_{0}^{2}-{z}^{2}}}\,\sin \,z).$$

**Figure 2:**
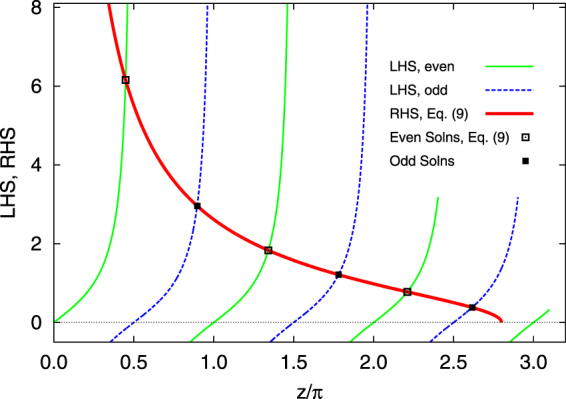
Graphical solution of Eq. (9), for an example *z*_0_ = 2.8*π*. The LHS is shown with the thin solid (green) curves, with the obvious characteristic branches of the tan function. The thicker solid (red) curve represents the RHS. The intersections of these two curves represent the *even* bound states and are indicated by open squares; the lowest energy solution is just below ${\tilde{z}}_{1}=\pi /2$. For completeness we have also drawn the LHS and RHS for the odd bound states. The LHS is given by $\tan ({\tilde{z}}_{1}-\pi /2)$ and is shown with thin dashed (blue) curves. The RHS is the same solid (red) curve as for the even states. Their intersections are indicated by filled squares. For our tight binding solutions we will focus on the lowest energy (even) bound state, i.e. the point with ${\tilde{z}}_{1}\mathop{ < }\limits_{ \tilde {}}\pi /2$.

Eq. (5) is still exact; now we can imagine the scenario where $b/\ell$ is very large, and hence the RHS of this equation is very small. The zeroth order solution for the even bound states is given by setting the first factor on the LHS of Eq. (5) to zero,

8 $$\cos \,{\tilde{z}}_{1}-\frac{{\tilde{z}}_{1}}{\sqrt{{z}_{0}^{2}-{\tilde{z}}_{1}^{2}}}\,\sin \,{\tilde{z}}_{1}=0,$$

and this determines the zeroth order solution, ${\tilde{z}}_{1}$. The equation to determine ${\tilde{z}}_{1}$ can be written as

9 $$\tan \,{\tilde{z}}_{1}=\sqrt{{(\frac{{z}_{0}}{{\tilde{z}}_{1}})}^{2}-1},$$

which is the equation that determines the even bound state energies for a particle in a *single* well of width *w* and depth *V*_0_. The solution is shown graphically in Fig. 2. An actual number for ${\tilde{z}}_{1}$, slightly less than *π*/2, is readily obtained for the lowest bound state, either numerically or on a calculator.

### First-order in tunneling

A more accurate solution to Eq. (5) can be obtained to 1^st^ order in *η*_1_(*k*) by writing $z={\tilde{z}}_{1}[1+\tilde{\rho }(k)]$, and expanding that equation to 1^st^ order in $\tilde{\rho }(k)$. After some algebra (see Appendix C) we obtain

10 $$\tilde{\rho }(k)=-\frac{2}{{z}_{0}^{2}}\frac{({z}_{0}^{2}-{\tilde{z}}_{1}^{2})}{(1+\sqrt{{z}_{0}^{2}-{\tilde{z}}_{1}^{2}})}{e}^{-\tfrac{2b}{w}\sqrt{{z}_{0}^{2}-{\tilde{z}}_{1}^{2}}}\,\cos \,k\ell .$$

**Figure 3:**
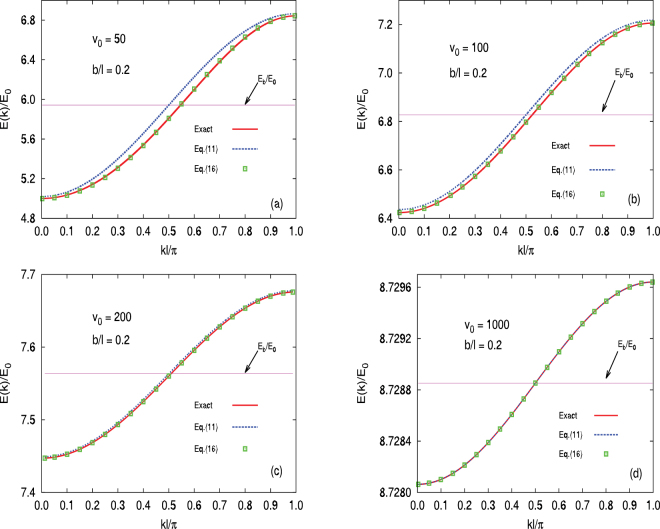
Comparison of various approximations with the exact result (solid (red) curve). All results are for $b/\ell =0.2$ and are for (**a**) *v*_0_ = 50, (**b**) *v*_0_ = 100, (**c**) *v*_0_ = 200, and (**d**) *v*_0_ = 1000. In (**a**), for example, the first-order result is given by Eq. (11) (shown as the dashed (blue) curve) with the exact result determined numerically from Eq. (1) (shown as the solid (red) curve). Note that there remains a significant discrepancy. The zeroth-order result, a constant given by the first term only in Eq. (11), is shown as the horizontal (pink) line [e.g. at *E*_*b*_/*E*_0_ ≈ 5.94 in (**a**)]. With further second-order corrections that arise from *η*_2_ and the nonlinear nature of Eq. (5), we also show the results from Eq. (16). This result now agrees very well with the exact result, and includes terms corresponding to −$2{t}_{2}\,\cos \,\mathrm{(2}k\ell )$ in Eq. (2). Notice that for the last case, all curves and points are essentially in agreement. Given the reduction in the scale of the bandwidth in (*a*) → (*d*), this is impressive agreement.

Note that (for now) we have ignored *η*_2_, as that factor is exponentially suppressed with respect to *η*_1_(*k*). Using the energy scale *E*_0_ = *ħ* ^2^/(2*mw* ^2^) as before, we find for the energy to 1^st^ order in *η*_1_(*k*),

11 $$\frac{E}{{E}_{0}}=4{\tilde{z}}_{1}^{2}-{(\frac{4{\tilde{z}}_{1}}{{z}_{0}})}^{2}\frac{{z}_{0}^{2}-{\tilde{z}}_{1}^{2}}{1+\sqrt{{z}_{0}^{2}-{\tilde{z}}_{1}^{2}}}{e}^{-2\tfrac{b}{w}\sqrt{{z}_{0}^{2}-{\tilde{z}}_{1}^{2}}}\,\cos \,k\ell $$

which has the functional form of nearest-neighbour tight-binding (see Eq. (2)). A comparison of this result (dotted blue curve) with the exact result (solid red curve) is shown in Fig. 3 as a function of wave vector for particular values of *V*_0_ and $b/\ell$.

If we first focus on case (a) in Fig. 3, the first-order result has significant disagreement with the exact result. It is indeed true that improved agreement is readily attained by using deeper wells, as is clear from the progression through (b–d). However, as we now illustrate, more accurate solutions are achievable by including contributions from *η*_2_, which is already 2^nd^ order in *e* ^−*x*^, where $x\equiv 2\tfrac{b}{w}\sqrt{{z}_{0}^{2}-{\tilde{z}}_{1}^{2}}$—see Eq. (7).

### Second-order in tunneling

We first repeat the steps leading to Eq. (10), i.e. we write the solution as $z\approx {\tilde{z}}_{1}(1+\tilde{\rho }(k))$, but now expand to 2^n*d*^ order in $\tilde{\rho }(k)$ as well as in *e* ^−*x*^, since *η*_2_/*η*_1_(*k*) ≈ *O*(*e* ^−*x*^). A straightforward calculation gives

12 $$-\tilde{\rho }(k)\frac{{z}_{0}}{{f}_{1}}\{1-\tilde{\rho }(k){g}_{1}\}=2{e}^{-x}\,\cos \,k\ell +2{z}_{0}\frac{2b}{w}{e}^{-x}\tilde{\rho }(k)\frac{{\tilde{\delta }}^{2}}{\sqrt{1-{\tilde{\delta }}^{2}}}\,\cos \,k\ell +2{e}^{-2x}(1-2{\tilde{\delta }}^{2}),$$

where

13 $$\begin{array}{rcl}{f}_{1} & = & \frac{1-{\tilde{\delta }}^{2}}{\sqrt{1-{\tilde{\delta }}^{2}}+\tfrac{1}{{z}_{0}}}\\ {g}_{1} & = & 1-\frac{{\tilde{\delta }}^{2}}{1-{\tilde{\delta }}^{2}}\frac{\sqrt{1-{\tilde{\delta }}^{2}}+\tfrac{3}{2{z}_{0}}}{\sqrt{1-{\tilde{\delta }}^{2}}+\tfrac{1}{{z}_{0}}},\end{array}$$

and $\tilde{\delta }\equiv {\tilde{z}}_{1}/{z}_{0}$. Eq. (12) is a quadratic in $\tilde{\rho }(k)$; since this is a small quantity we can simply iterate to obtain $\tilde{\rho }(k)$ explicitly:

14 $$\begin{array}{rcl}\tilde{\rho }(k) & = & -\frac{2}{{z}_{0}}{f}_{1}{e}^{-x}\,\cos \,k\ell \\  &  & -\frac{2{f}_{1}}{{z}_{0}}{e}^{-2x}\,(1-2{\tilde{\delta }}^{2}-\frac{{f}_{1}}{{z}_{0}}\,({g}_{1}+{z}_{0}\frac{2b}{w}\frac{{\tilde{\delta }}^{2}}{\sqrt{1-{\tilde{\delta }}^{2}}}))\\  &  & +\frac{2{f}_{1}^{2}}{{z}_{0}^{2}}{e}^{-2x}\,({g}_{1}+{z}_{0}\frac{2b}{w}\frac{{\tilde{\delta }}^{2}}{\sqrt{1-{\tilde{\delta }}^{2}}})\,\cos \,2k\ell .\end{array}$$

Finally, we use

15 $$E(k)=4{E}_{0}{\tilde{z}}_{1}^{2}{\mathrm{(1}+\tilde{\rho })}^{2}$$

from which we obtain

16 $$E(k)={E}_{c}-2{t}_{1}\,\cos \,k\ell -2{t}_{2}\,\cos \,2k\ell ,$$

where the parameters defined by Eq. (16) are given by

17 $${t}_{1}=8{E}_{0}{z}_{0}{\tilde{\delta }}^{2}{f}_{1}{e}^{-x},$$

18 $${t}_{2}=-8{E}_{0}{\tilde{\delta }}^{2}{f}_{1}^{2}\,({g}_{1}+\frac{1}{2}+{z}_{0}\frac{2b}{w}\frac{{\tilde{\delta }}^{2}}{\sqrt{1-{\tilde{\delta }}^{2}}})\,{e}^{-2x}$$

and

19 $${E}_{c}={E}_{0}\{4{\tilde{z}}_{1}^{2}-16{\tilde{z}}_{1}\tilde{\delta }{f}_{1}{e}^{-2x}(1-2{\tilde{\delta }}^{2})\}-2{t}_{2}.$$

Note that the expansion is governed by the exponential suppression contained in the *e* ^−*x*^ and *e* ^−2*x*^ factors. However, we expect *z*_0_ > 1 for tight-binding, whereas Fig. 2 makes it clear that ${\tilde{z}}_{1}$ is of order unity or lower; more precisely, ${\tilde{z}}_{1} < \,{\rm{\min }}({z}_{0},\pi /2)$, and so $\tilde{\delta } < 1$ as well. We have written the above expressions to make these expansion parameters clear; thus, even within the expression for *t*_2_, for example, various terms will contribute significantly less than others. Both *f*_1_ and *g*_1_ are of order unity.

## Discussion

The more accurate result from Eq. (16) is plotted in Fig. 3 (as indicated by the square symbols), and gives good agreement for the parameters used in that figure. Note that *t*_1_ is given by an expression identical to the one implied in Eq. (11)—by going to 2^nd^ order in *e* ^−*x*^ this has not changed, and in fact is identical to the expression derived for the double-well in Appendix A, Eq. (A5). The need to to go to 2^nd^ order and therefore generate a term with $\cos \,\mathrm{(2}k\ell )$ wave vector dependence is sometimes interpreted to mean that a significant tunneling amplitude exists between second-nearest-neighbour atoms. The fact that this is required even for the case of the double-well studied in Appendix A (where there is no second-nearest neighbour!) indicates that this interpretation is incorrect. Indeed it is a difficult problem to disentangle contributions to 2^nd^ order from next-nearest-neighbour tunneling and contributions arising from the inherent non-linear nature of the equations; at this point we simply caution that all these contributions are not entirely due to direct next-nearest-neighbour tunneling.

**Figure 4:**
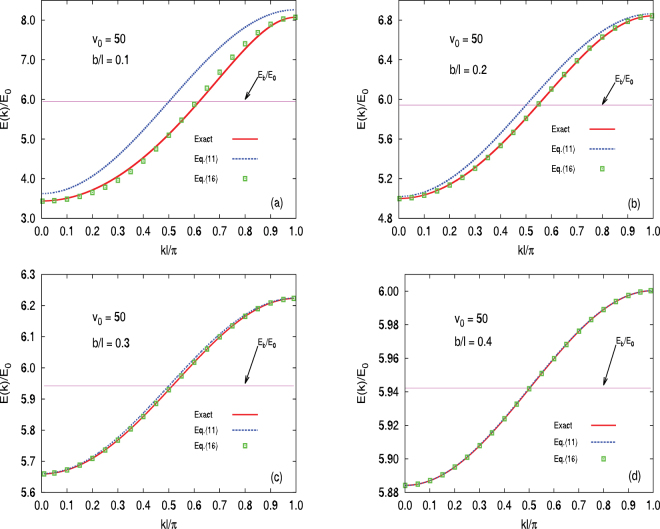
As in Fig. 3, a comparison of various approximations with the exact result (solid (red) curve) for a variety of barrier widths, all with *v*_0_ ≡ *V*_0_/*E*_0_ = 50. We use barrier widths of (**a**) $b/\ell =0.1$, (**b**) $b/\ell =0.2$, (**c**) $b/\ell =0.3$ and (**d**) $b/\ell =0.4$. By examining the vertical scales, a clear progression towards more tightly bound wells is evident. Moreover, the first case considered shows not perfect agreement, even when 2^nd^ order corrections are included. As $b/\ell$ increases, agreement quickly improves, especially when account is made of the steady reduction in the scale of the bandwidth. For all cases, the horizontal (pink) line corresponds to the bound state energy for a single well and corresponds to the constant given by the first term only in Eq. (11). The approximate result with the 1^st^ order correction only, the full Eq. (11), is shown as a dashed (blue) curve, while the approximate results including 2^nd^ order corrections from Eq. (16) are given by the square (green) symbols.

For completeness, in Fig. 4 we fix the well depth to be *v*_0_ ≡ *V*_0_/*E*_0_ = 50 and show the dispersions for a variety of different barrier widths. The trends are the same in the two cases, except that the 2^nd^ order result is slightly less accurate for the least tightly bound case considered [see (a)]. With increasing barrier width, however, as in Fig. 3, both the 2^nd^-order and the 1^st^-order results become increasingly accurate. Note the change in scale as *v*_0_ and $b/\ell$ increase in Figs 3 and 4, respectively; in both cases the results approach the single well result while a well-defined dispersion remains.

**Figure 5:**
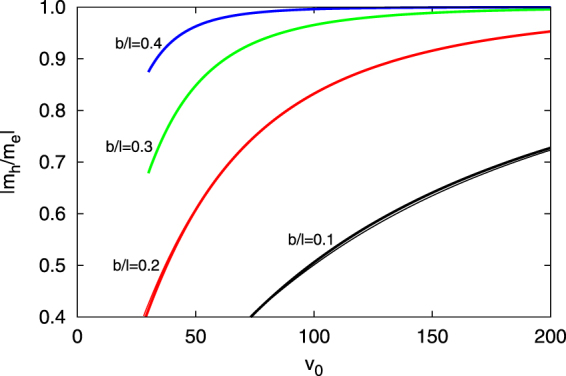
Absolute value of the effective mass ratio, |*m*_*h*_/*m*_*e*_| vs. *v*_0_ ≡ *V*_0_/*E*_0_ for the various values of barrier width $b/\ell$, as indicated. Here ${m}_{e}^{-1}\equiv \tfrac{{\partial }^{2}E(k)/{E}_{0}}{\partial {(k\ell )}^{2}}$ at *k* = 0 and ${m}_{h}^{-1}\equiv \tfrac{{\partial }^{2}E(k)/{E}_{0}}{\partial {(k\ell )}^{2}}$ at $k=\pi /\ell$. The thick curves are the exact result, determined from Eq. (1), while the thinner curves are determined from the tight-binding parametrization of Eq. (16). These latter curves are barely visible over almost the entire parameter regime shown, indicating that when the 2nd order corrections considered are included the derived tight-binding parameters are very accurate.

In both Figs 3 and 4 it should be clear that for the more strongly coupled wells (e.g. (a) and (b) in particular) a significant amount of electron-hole asymmetry is present. In Fig. 5 the effective mass ratio, |*m*_*h*_/*m*_*e*_| is shown for various values of the barrier thickness as a function of well depth. Here, the electron mass, *m*_*e*_ is defined in the usual way (see the caption in Fig. 5) through the curvature at *k* = 0 and similarly for the hole mass, *m*_*h*_. As discussed in ref.^6^ an asymmetry is expected on general grounds since holes are by definition closer to the top of the barriers than electrons. They should therefore have lower masses for this reason alone, and this is reflected in the results of Fig. 5, where all the ratios are lower than unity. The thicker curves are from the exact calculations while the thinner curves (in too good agreement with the exact results to be visible for most of the parameter space shown) are readily determined from the tight-binding parametrization of Eq. (16). These are fairly accurate when the higher-order correction considered above is included.

Figure 5 exemplifies what is perhaps the primary result of this calculation—that electron-hole asymmetry is the “rule”, not the exception. In the past three decades, as more materials with exotic properties (superconductivity, magnetoresistance, etc.) have been engineered, particularly through doping, and electron-hole-doping symmetry has *not* been observed, strong correlations are often suggested as a possible explanation. The present work notes that the usual starting point, tight-binding with nearest-neighbour hopping only, tends to bias the expectation in favour of electron-hole symmetry, when in fact the opposite is true. Our calculations illustrate that even where merely nearest-neighbour hopping seems like a good physically-motivated assumption, the band structure (before invoking interaction terms) will be already electron-hole asymmetric. For example, Hirsch and one of the present authors^11^ suggested a modulated hopping model in which one of the interactions is explicitly particle-hole asymmetric in a tight-binding framework (see ref.^12^ for an overview of the physics of hole superconductivity). It follows that many of the properties of this model will exhibit electron-hole asymmetries. An accurate confrontation with experiment will required some knowledge of the asymmetry already possibly present in the band structure, not necessarily because of a next-nearest-neighbour hopping term, but for the reasons suggested in this paper—the smaller tunneling barrier faced by holes compared to electrons.

## Summary

We have succeeded in deriving the effective model for the periodic potential first used to model a solid, the so-called Kronig-Penney model, consisting of a series of wells and barriers. As is more readily illustrated for the double-well potential, one can achieve very high accuracy by exploiting the tightly-bound limit, where two neighbouring wells are well separated. This ensures that the tunneling amplitude between the two wells is very small, and one can essentially use perturbation theory with respect to this “atomic limit”.

The generalization of this process to an infinite array of wells and barriers is straightforward. However, incorporating tunneling to 1^st^ order (meaning terms of order *e* ^−*x*^, where $x\propto b\sqrt{{V}_{0}}$) provided only a *qualitative* agreement with the exact result (this would become quantitative for sufficiently large $b/\ell$ and/or *V*_0_). When including terms of 2^nd^ order in *e* ^−*x*^, very good quantitative agreement was achieved, even for moderate well depths. Terms of order *e* ^−2*x*^ would necessarily be accompanied by dispersive terms like $\cos \,\mathrm{(2}k\ell )$, which are generally associated with next-nearest-neighbour tunneling, i.e. tunneling across two barriers. By comparison with results of a simple double-well, where terms of order *e* ^−2*x*^ also contribute to the energy, we were able to show that terms of this order were not exclusively associated with such longer-range tunneling. Instead, inherent non-linearity of the equations governing the electronic energy dispersion will naturally give rise to such terms, even in the absence of next-nearest-neighbour tunneling.

It is useful to map the complete microscopic double-well problem onto the two-state system that is often used to describe this problem in simplified terms. Similarly, it is useful to map the microscopic problem of an infinite array of wells onto a simplified model—this is the tight-binding description. We have carried out such a mapping, with no “fitting” involved and we have illustrated the accuracy as well as the limitations of such a mapping.

## Electronic supplementary material

Supplementary Information
