# Supplementary material for: The tight-binding formulation of the Kronig-Penney model
Source: Sci Rep. 2017 Dec 6;7:17041. doi: 10.1038/s41598-017-17223-2 (PMC5719031; doi:10.1038/s41598-017-17223-2)
Supplement: Supplementary file 1 — Supplementary Information [file 41598_2017_17223_MOESM1_ESM.pdf]

The tight-binding formulation of the Kronig-Penney model: Appendices

F. Marsiglio and R. L. Pavelich*
Department of Physics, University of Alberta, Edmonton, AB, Canada T6G 2E1

(Dated: November 22, 2017)

Appendix A: The Kronig-Penney model reduced to two cells: a double-well potential

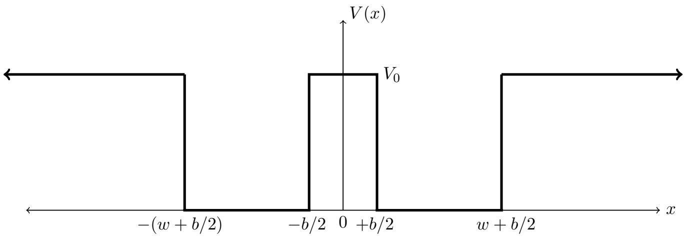

Fig A1: A simple double-well potential, consisting of two wells, each of depth V_0 and width w, separated by a barrier of height V_0 and width b. Unlike the potential depicted later in Appendix B (Fig. B1), the potential of height V_0 extends to $x \to \pm\infty$ beyond the double-well region.

This simple double-well potential consists of two wells separated by a barrier of height V_0 and width b, i.e. two cells of Fig. 1, but with infinitely long "flat" regions extending out to $x \to \pm\infty$. Moreover, these wells are centered around $x = 0$ and each have width w. The analytical form is

$$V_{\rm sq}(x) = \begin{cases} 0 & \text{if } \left| |x| - (\frac{b+w}{2}) \right| < \frac{w}{2} \\ V_0 & \text{otherwise;} \end{cases} \tag{A1}$$

this potential is illustrated in Fig. A1. Note that the form of this potential is identical to that in Ref. [3] but our definitions of certain parameters differ from those used there. Bound state solutions are categorized as either even or odd about the central barrier. A piecewise-continuous wave function is required over five different regions; then a straightforward matching of the wave function and its derivative at the potential discontinuities yields the result

$$\left(\cos\frac{qw}{2} - \frac{q}{\kappa_2}\sin\frac{qw}{2}\right)\left(\cos\frac{qw}{2} + \frac{\kappa_2}{q}\sin\frac{qw}{2}\right) = \pm\tilde{\eta} \tag{A2}$$

where, as in the body of this paper, $q \equiv \sqrt{2mE/\hbar^2}$ and $\kappa_2 \equiv \sqrt{2m(V_0 - E)/\hbar^2}$. The positive (negative) sign refers to the even (odd) parity solution. This result is very similar to Eq. (3) except here the RHS can take on only two values, with $\tilde{\eta} \equiv k_0^2 \sin(qw/2)\cos(qw/2)\, e^{-\kappa_2 b}$. Now when there is no coupling between the wells (i.e. $b \to \infty$ and therefore $\tilde{\eta} \to 0$), then the vanishing of the first (second) factor on the LHS corresponds to determining the energy for the even (odd) bound states in the single well, as was the case in Eq. (3). Using the same dimensionless parameters as in the Kronig-Penney case, we define $z \equiv qw/2$ and $z_0 \equiv k_0 w/2$, where $k_0 \equiv \sqrt{2mV_0/\hbar^2}$. Then Eq. (A2) becomes

$$\left(\cos z - \frac{z}{\sqrt{z_0^2 - z^2}}\sin z\right)\left(\cos z + \frac{\sqrt{z_0^2 - z^2}}{z}\sin z\right) = \pm\frac{z_0^2}{z_0^2 - z^2}\sin z\cos z\; e^{-\frac{2b}{w}\sqrt{z_0^2 - z^2}} \tag{A3}$$

where we have used $\kappa_2 w/2 = \sqrt{z_0^2 - z^2}$.

As in the Kronig-Penney case, the zeroth order solution is given by $\tilde{z}_1$ (see Eq. (8) or (9)), and the solution to Eq. (A3) can be obtained to $1^{\rm st}$ order in $\tilde{\eta}$ by writing $z = \tilde{z}_1 + \tilde{\rho}$. With algebra similar

to that which produced Eq. (10), we obtain a result very similar to that equation:

$$\tilde{\rho} = \mp \frac{\tilde{z}_1}{z_0^2} \frac{(z_0^2 - \tilde{z}_1^2)}{(1 + \sqrt{z_0^2 - \tilde{z}_1^2})} e^{-\frac{2b}{w}\sqrt{z_0^2 - \tilde{z}_1^2}}. \tag{A4}$$

As expected, the negative (positive) result is precisely *half* the value give by Eq. (10) with $k = 0$ ($k = \pi/\ell$), a result well known for tight-binding models when only two sites (without periodic boundary conditions) are used.

Therefore a toy model with two states only, corresponding to "particle in left well" and "particle in right well", each with energy $E_b = 4\tilde{z}_1^2 E_0$ (see Eq. (11)), that has a tunneling amplitude $\tilde{t}$ for one of these two degenerate states to tunnel into the other then results in two states with non-degenerate energies, $E_b \mp \tilde{t}$. The parameter $\tilde{t}$ is given by the same value as in the Kronig-Penney model, Eq. (17), reproduced here for convenience:

$$\tilde{t} = t_1 = 8E_0\tilde{z}_1^2 \frac{1 - \tilde{\delta}^2}{1 + z_0\sqrt{1 - \tilde{\delta}^2}} e^{-2\frac{b}{w}z_0\sqrt{1-\tilde{\delta}^2}} = 8E_0 z_0 \tilde{\delta}^2 f_1 e^{-x}, \tag{A5}$$

with $\tilde{\delta} \equiv \tilde{z}_1/z_0$, $x \equiv 2\frac{b}{w}\sqrt{z_0^2 - \tilde{z}_1^2}$, and

$$f_1 = \frac{1 - \tilde{\delta}^2}{\sqrt{1 - \tilde{\delta}^2} + \frac{1}{z_0}}. \tag{A6}$$

It is clear that this double-well potential (as opposed to the one discussed in Appendix B more naturally generalizes to the Kronig-Penney model described in Sec. II.

Since good agreement with the exact results will be seen to require higher-order corrections (see Sec. II and Appendix B), we state the result here as well. With

$$E_\pm = E_b^{(2)} \mp \tilde{t} \tag{A7}$$

with the superscript '(2)' referring to the fact that $2^{\rm nd}$ order corrections in e^{-x} are now included, we find that $\tilde{t}$ is unchanged from the previous result [Eq. (A5)], but the base term, $E_b^{(2)}$, becomes

$$E_b^{(2)} = 4\tilde{z}_1^2 \left(1 - \frac{2f_1^2}{z_0} e^{-2x} f_2\right), \tag{A8}$$

where

$$f_2 = \frac{1 - 2\tilde{\delta}^2}{\sqrt{1 - \tilde{\delta}^2}} - \frac{1}{2z_0} - \frac{2b}{w}\frac{\tilde{\delta}^2}{\sqrt{1 - \tilde{\delta}^2}} + \frac{1}{z_0^4}\frac{\tilde{\delta}^2}{1 - \tilde{\delta}^2}\frac{1 - z_0^2\tilde{\delta}^2/2}{\sqrt{1 - \tilde{\delta}^2} + \frac{1}{z_0}}. \tag{A9}$$

Note that the first term in f_2 is of order unity but subsequent terms are of lower order in $1/z_0$. Fig. A2 shows the two split (lowest) energies for three examples ($b = 0.1$, $b = 0.2$ and $b = 0.3$, with $w \equiv 1 - b$ to conform with the Kronig-Penney parameters). Note that the energies with $2^{\rm nd}$ order corrections are in good agreement for essentially all values of V_0 for all three cases, whereas the energies calculated with $1^{\rm st}$-order corrections only agree with the exact solutions only in case (c) or for larger values of v_0 than shown here. All of these higher order corrections occur due to the inherent nonlinear nature of Eq. (A3), i.e. they do *not* occur because of next-nearest-neighbour tunneling (since there are only two wells and hence no next-nearest neighbours).

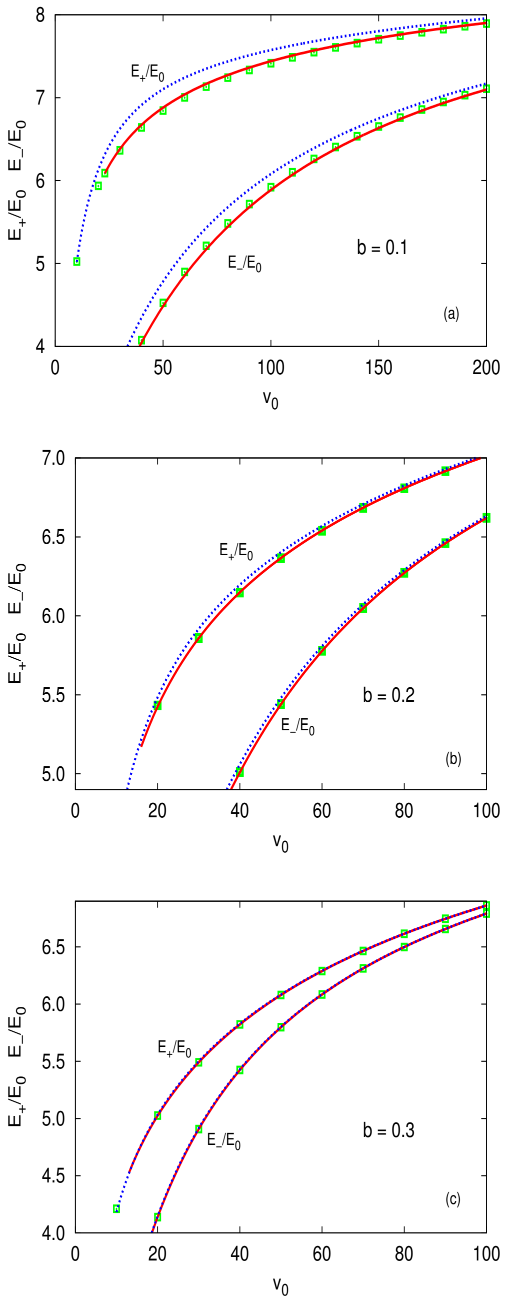

Fig A2: Comparison of the first-order result given by $E_{\pm} = E_b \pm \tilde{t}$ (dashed blue curve) and the 2$^{\rm nd}$-order result given by Eq. (A8) (green squares) with the exact result determined numerically from Eq. (A3) (solid red curve) as a function of $v_0 \equiv V_0/E_0$ for (a) $b = 0.1$, (b) $b = 0.2$, and (c) $b = 0.3$, with the well width adjusted so that $w = 1 - b$ (to conform with the Kronig-Penney case treated in Sec. II and in Appendix B). The 2$^{\rm nd}$-order solution is accurate in all three cases, but the 1$^{\rm st}$-order solution is accurate only when the two wells are sufficiently far apart [case (c), where essentially all of the curves and points agree].

Appendix B: Double-well Potential of Ref. [2]

This double-well potential was specifically discussed in Ref. [2]; a two-state model was fitted there, and an approximate (WKB) approximation yielded imperfect parameters. Here in contrast, we provide a systematic derivation of these parameters; for the values used in Ref. [2] we get perfect agreement with those obtained there through fitting. As illustrated in Fig. B1, this double-well

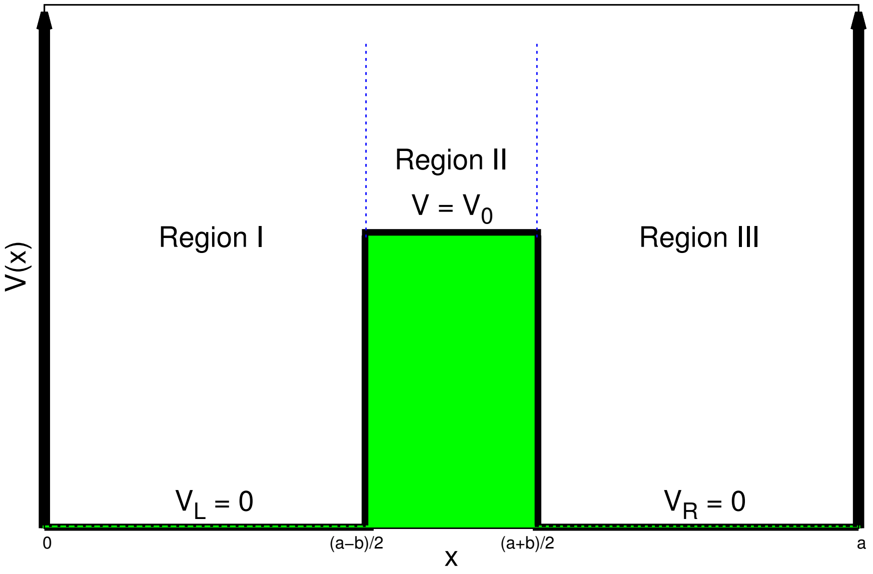

Fig B1: Schematic of the double-well potential used in Ref. [2].

potential is defined by

$$V(x) = \begin{cases} \infty & \text{if } x < 0 \text{ or } x > a \\ V_0 & \text{if } (a-b)/2 < x < (a+b)/2 \\ V_L & \text{if } 0 < x < (a-b)/2 \\ V_R & \text{if } (a+b)/2 < x < a. \end{cases} \tag{B1}$$

This potential describes two wells, each of width $w \equiv (a-b)/2$, separated by a barrier of width b and height V_0. The "floor" level of each well is in principle variable, but here we consider only the symmetric case, given by $V_L = V_R = 0$. A straightforward solution, valid for $E < V_0$, is

$$\begin{aligned} \psi_I(x) &= A \sin qx & q &\equiv \sqrt{2mE/\hbar^2} \\ \psi_{II}(x) &= Be^{\kappa_2 x} + Ce^{-\kappa_2 x} & \kappa_2 &\equiv \sqrt{2m(V_0-E)/\hbar^2} \\ \psi_{III}(x) &= D \sin q(a-x) \end{aligned} \tag{B2}$$

where the regions I, II, and III refer to $0 < x < w$, $w < x < w+b$, and $w+b < x < a$, respectively. If we separately adopt suitable parameters for even and odd solutions (with respect to $x = a/2$), then matching the wave functions and their derivatives at the boundaries leads to

$$\tan(2z - \pi/2) = \left[\sqrt{\left(\frac{z_0}{z}\right)^2 - 1}\right] \tanh\left(\frac{b}{w}\sqrt{z_0^2 - z^2}\right) \tag{B3}$$

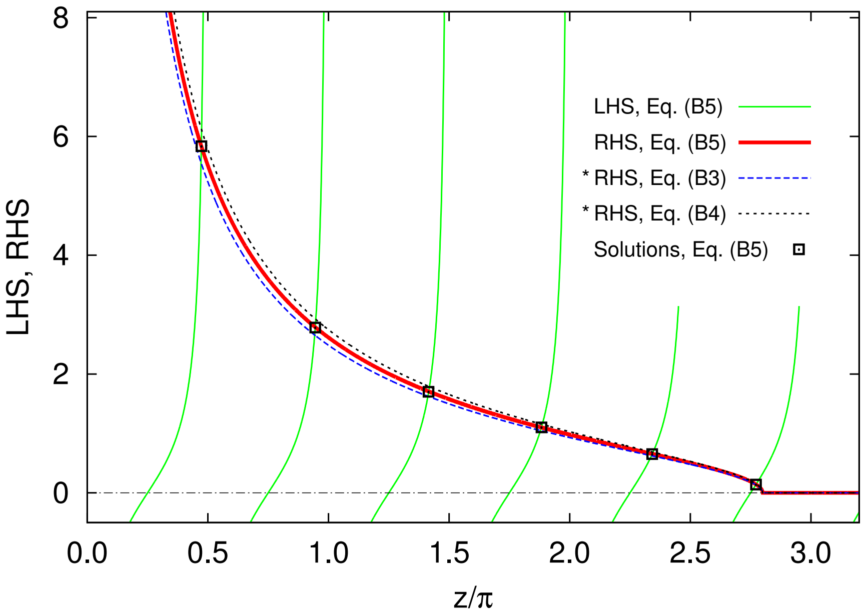

Fig B2: Graphical solution of Eq. (B3-B5), for an example $z_0 = 2.8\pi$. The LHS is shown with the thin solid (green) curves with the obvious characteristic branches of the tan function. The thick solid (red) curve represents the RHS and lies centrally between two other curves. Intersections of LHS and RHS represent solutions to Eq. (B5), with the lowest energy solution just below $z_1 = \pi/2$; these solutions are indicated by squares in the figure. The two thinner curves bracketing the thick solid (red) curve are the RHSs of Eq. (B3) [(blue) dashed curve slightly lower] and of Eq. (B4) [(black) dotted curve slightly higher]. The asterisks on the labels indicate that the deviation from the central (red) curve has been exaggerated for clarity. The intersection of these curves with the thin green curve indicates a slight energy lowering and energy raising, respectively, with respect to the single well solution.

for the even solution, and

$$\tan(2z - \pi/2) = \left[\sqrt{\left(\frac{z_0}{z}\right)^2 - 1}\right] \coth\left(\frac{b}{w}\sqrt{z_0^2 - z^2}\right) \tag{B4}$$

for the odd solution, where $z \equiv qw/2$ and $z_0 \equiv k_0 w/2$, with $k_0 \equiv \sqrt{2mV_0/\hbar^2}$. If we use energy units $E_0 \equiv \hbar^2/(2mw^2)$, then $E/E_0 = 4z^2$. For a single well, i.e. with $b/w \to \infty$, the hyperbolic functions are unity and the energy is given by the solution of the simpler equation,

$$\tan(2z_1 - \pi/2) = \sqrt{\left(\frac{z_0}{z_1}\right)^2 - 1}; \tag{B5}$$

we use z_1 do denote this single well solution, and presume that it is obtained numerically or on a calculator by iteration. The energy corresponding to this level will be denoted $E_1 = 4z_1^2 E_0$. It is not hard to see that Eq. (B3) results in a slightly lower energy solution (compared to the energy E_1, the solution of Eq. (B5)), while Eq. (B4) results in a slightly higher energy solution. Mathematically this

is because the hyperbolic tangent is always slightly less than unity while the hyperbolic cotangent is always slightly greater than unity. Physically this corresponds to the bonding and anti-bonding solutions to a particle which is given freedom to move in two basins (i.e. an electron free to roam among two atoms in a molecule). The situation is illustrated graphically in Fig. B2; the left-hand-sides (LHSs) are the same in all three equations, and are indicated by the solid (green) curves. The right-hand-side (RHS) of Eq. (B5) is indicated by the thick solid (red) curve that lies in between the two curves representing the RHSs of Eq. (B3) (in blue, below) and Eq. (B4) (in black, above), with a slightly lower and higher energy, respectively. This fine splitting of an otherwise degenerate level is what is expected for a significant barrier between the two wells. As stated earlier, generically one expects that coupling N wells will result in a splitting into N energies.

Let us focus on the most tightly bound, lowest, energy level. Then the argument in the hyperbolic functions will be very close to unity; expanding to first order results in

$$\tan(2z - \pi/2) = \sqrt{\left(\frac{z_0}{z}\right)^2 - 1}\left[1 \mp 2\,\exp\left\{-2\frac{b}{w}\sqrt{z_0^2 - z^2}\right\}\right], \tag{B6}$$

where the minus [plus] sign results from Eq. (B3) [B4], and the exponential correction is expected to be very small. We thus look for solutions

$$z_{e,o} = z_1 \mp \rho, \tag{B7}$$

where, as mentioned above, z_1 is presumed known (and somewhat less than $\pi/2$), and the subscript 'e' ('o') corresponds to the even (odd) solution. Inserting Eq. (B7) into Eq. (B6), and expanding everywhere to first order in ρ results in

$$\rho = 2\delta z_0 \frac{1-\delta^2}{1+2z_0\sqrt{1-\delta^2}} \exp\left\{-2\frac{b}{w}z_0\sqrt{1-\delta^2}\right\}, \tag{B8}$$

where δ is the single-well energy level, $\delta \equiv z_1/z_0 = \sqrt{E_1/V_0}$, determined in advance.

If we use the values from Ref. [2], i.e. $V_0 = 500\pi^2\hbar^2/(2ma^2) = 500\pi^2 E_0 w^2/a^2 = 80\pi^2 E_0$ for $w/a = 2/5$, then $z_0 = \pi\sqrt{80}$. We can solve Eq. (B5) on a calculator, and we obtain $\delta \approx 0.108$. Plugging this into Eq. (B8) we find $\rho \approx 1.78 \times 10^{-7}$.

The "toy model" here is a two-state system, as in the Feynman example, but with a wave function describing the particle to be in the left well (ψ_L) and a wave function for the particle in the right well (ψ_R). The tunneling amplitude t mentioned in the Introduction and defined in Eq. (11) of Ref. [2] as the matrix element for tunneling from the left well into the right well (or vice-versa), is defined by the correspondence between the energy there, $E = E_1 - t$, and the energy here, $E = 4z_1^2 E_0 - 8E_0\rho z_1$. More explicitly, we repeat here Eq. (11) from Ref. [2]:

$$\begin{aligned} H\psi_L &= E_1\psi_L - t\psi_R, \\ H\psi_R &= E_1\psi_R - t\psi_L, \end{aligned} \tag{B9}$$

which describes the coupling between the two states through the parameter t. Comparing to the expression above Eq. (B9) shows that $t \equiv 8E_0\rho z_1$. Therefore,

$$t = 16E_0 z_1^2 \frac{1-\delta^2}{1+2z_0\sqrt{1-\delta^2}} e^{-2\frac{b}{w}z_0\sqrt{1-\delta^2}}; \tag{B10}$$

with the parameters used above, we obtain

$$t \approx 1.08 \times 10^{-6} E_0, \tag{B11}$$

and in the units of Ref. [2], we have

$$t \approx 6.84 \times 10^{-7} \frac{\pi^2 \hbar^2}{2ma^2}, \tag{B12}$$

which is precisely what was obtained there through a fit to the numerical data.

In summary, we have obtained the toy model parameter t, which describes the transition amplitude for the particle to tunnel from the left side of the double-well to the right side (or vice-versa), in terms of characteristics of the microscopic model and parameters involving the single well. In Ref. [2] a qualitative estimate was provided, based on a WKB approximation. Here we have improved considerably on this estimate, and now have a quantitatively accurate correspondence between the "microscopic" double-well potential and the two-state system.

Appendix C: Derivation of Eq. (10)

We begin with Eq. (5), but with the higher order correction, η_2 omitted. Then,

$$\left(\cos z - \frac{z}{\sqrt{z_0^2 - z^2}} \sin z\right)\left(\cos z + \frac{\sqrt{z_0^2 - z^2}}{z} \sin z\right) \approx 2e^{-2\frac{b}{w}\sqrt{z_0^2 - \tilde{z}_1^2}} \cos k\ell. \tag{C1}$$

Since the right-hand-side (RHS) is exponentially small, and we will pursue the dispersion for the lowest (even) bound state, then the solution is given by

$$z = \tilde{z}_1 \left[1 + \tilde{\rho}(k)\right], \tag{C2}$$

where $\tilde{\rho}(k)$ is a small relative correction to the solution for a single well, denoted by $\tilde{z}_1$, and determined by the first factor on the left-hand-side (LHS) of Eq. (C1) being zero. That is, Eq. (8) determines $\tilde{z}_1$.

Inserting the solution given by Eq. (C2) and expanding the first factor of Eq. (C1) to first order in $\tilde{\rho}(k)$, gives for this first factor

$$\left(\cos z - \frac{z}{\sqrt{z_0^2 - z^2}} \sin z\right) \approx -\tilde{\rho}(k)\left(\sqrt{z_0^2 - \tilde{z}_1^2} + 1\right)\frac{z_0^2 \cos \tilde{z}_1}{z_0^2 - \tilde{z}_1^2}. \tag{C3}$$

Since Eq. (C3) is already first order in $\tilde{\rho}(k)$ (as it must be), then the second factor on the LHS of Eq. (C1) is required only to zeroth order; we readily obtain

$$\left(\cos z + \frac{\sqrt{z_0^2 - z^2}}{z} \sin z\right) \approx \frac{z_0^2}{\tilde{z}_1^2} \cos \tilde{z}_1. \tag{C4}$$

Taking the product of Eq. (C3) and Eq. (C4) gives

$$\text{LHS} \approx -\tilde{\rho}(k)\left(\sqrt{z_0^2 - \tilde{z}_1^2} + 1\right)\frac{z_0^2}{z_0^2 - \tilde{z}_1^2}. \tag{C5}$$

where we have used $\cos^2 \tilde{z}_1 = \tilde{z}_1^2/z_0^2$. Equating this to the RHS of Eq. (C1) then gives Eq. (10).

---

* fm3@ualberta.ca, rpavelic@ualberta.ca

1 Feynman, R.P., Leighton, R. B., and Sands, M. The Feynman Lectures on Physics, Volume III (Addison-Wesley, Reading, MA, 1965).

2 Dauphinee, T., and Marsiglio, F., Asymmetric wave functions from tiny perturbations. Am. J. Phys. **83**, 861–866 (2015).

3 Jelic, V., and Marsiglio, F., The double-well potential in quantum mechanics: a simple, numerically exact formulation. Eur. J. Phys. **33**, 1651–1666 (2012).

4 Bloch, F., Über die Quantenmechanik der Elektronen in Kristallgittern. Z. Phys. **52**, 545–555 (1929), in German.

5 Ashcroft, N.W., and Mermin, N.D., Solid State Physics. 1st ed. (Brooks/Cole, Belmont, CA, 1976). See Appendix E.

6 Pavelich, R.L., and Marsiglio, F. The Kronig-Penney model extended to arbitrary potentials via numerical matrix mechanics. Am. J. Phys. **83**, 773–781, (2015).

7 Pavelich, R.L., and Marsiglio, F. Calculation of 2D electronic band structure using matrix mechanics. Am. J. Phys. **84**, 924–935 (2016).

8 Bloch, F., Memories of electrons in crystals. Proc. R. Soc. Lond. A **371**, 24–27 (1980).

9 See, for example, Nomura, Yusuke, Ab initio studies on superconductivity in alkali-doped fullerides. (Springer, Toronto, 2014).

10 Kronig, R. de L., and Penney, W. G., Quantum Mechanics of Electrons in Crystal Lattices. Proc. R. Soc. Lond. A, **130**, 499–513, (1931).

11 Hirsch, J. E. and Marsiglio, F., Superconducting state in an oxygen hole metal. Phys. Rev. **B 39**, 11515-11525, (1989).

12 Hirsch, J. E., Towards an understanding of hole superconductivity. High-Tc Copper Oxide Superconductors and Related Novel Materials, Vol. 255, 99-115 (2017), edited by Annette Bussmann-Holder, Hugo Keller, and Antonio Bianconi.
